# The Role of Biotics in Rosacea: A Narrative Review

**DOI:** 10.7759/cureus.105799

**Published:** 2026-03-24

**Authors:** Alondra L Avendaño-Pérez, Luz Orozco-Covarrubias, Marimar Saez-de-Ocariz

**Affiliations:** 1 Pediatric Dermatology, Instituto Nacional de Pediatría, Mexico City, MEX

**Keywords:** gastrointestinal microbiome, microbiota, prebiotics, probiotics, rosacea, skin microbiome, synbiotics

## Abstract

Rosacea is a chronic inflammatory dermatosis affecting the central face of adults, characterized by persistent erythema, flushing, papules, pustules, telangiectasias, and, in some cases, phymatous changes. Its pathogenesis involves a multifactorial interplay of immune dysregulation, neurovascular alterations, genetic predisposition, and disturbances of the skin and gut microbiota. Increasing interest in the gut-skin axis has prompted investigation into microbiota-targeted therapies, including probiotics, prebiotics, postbiotics, and synbiotics.

This narrative review evaluates current evidence regarding the role of biotics in rosacea. A literature search was conducted in PubMed/MEDLINE, Scopus, and Web of Science using combinations of the terms “rosacea,” “gut-skin axis,” “probiotics,” “prebiotics,” “postbiotics,” and “synbiotics.” Clinical trials, observational studies, and relevant mechanistic investigations published in English were considered.

Available data suggest that certain probiotic strains, administered orally or topically, may improve inflammatory lesions, erythema, and skin barrier function, particularly as adjuncts to standard therapy. However, findings are characterized by significant heterogeneity in strains, dosages, study design, outcome measures, and treatment duration. Evidence supporting the use of prebiotics, postbiotics, and synbiotics in rosacea remains limited and, in many cases, extrapolated from related inflammatory conditions or preclinical models.

Although microbiota modulation represents a promising therapeutic avenue, current evidence is insufficient to establish standardized clinical recommendations. Larger, well-designed randomized controlled trials with standardized endpoints and long-term follow-up are required to clarify their efficacy, optimal formulations, and safety.

## Introduction and background

General features of rosacea

Rosacea is a chronic inflammatory skin condition affecting about 5.5% of adults, mainly aged 30-50. While traditionally seen as more common in women, recent studies indicate similar prevalence in both sexes [1]. It primarily affects the central face (cheeks, nose, chin, and forehead) and follows a relapsing-remitting course. Key clinical features include flushing, burning or stinging sensations, persistent erythema, papules, pustules, telangiectasias, and sebaceous gland enlargement [2-4].

Rosacea has been didactically classified into erythematotelangiectatic, papulopustular, phymatous, and ocular subtypes [5]. However, shifting to a phenotype-based approach defines rosacea by at least one of two diagnostic features (persistent centrofacial erythema or phymatous changes) or two of four major features (flushing, telangiectasia, papules/pustules, or ocular signs); burning, stinging, edema, dryness, and some ocular findings are secondary phenotypes [6]. Common triggers such as stress, extreme temperatures, alcohol, and certain drugs can worsen symptoms [3,4].

Pathophysiology

**Figure 1:**
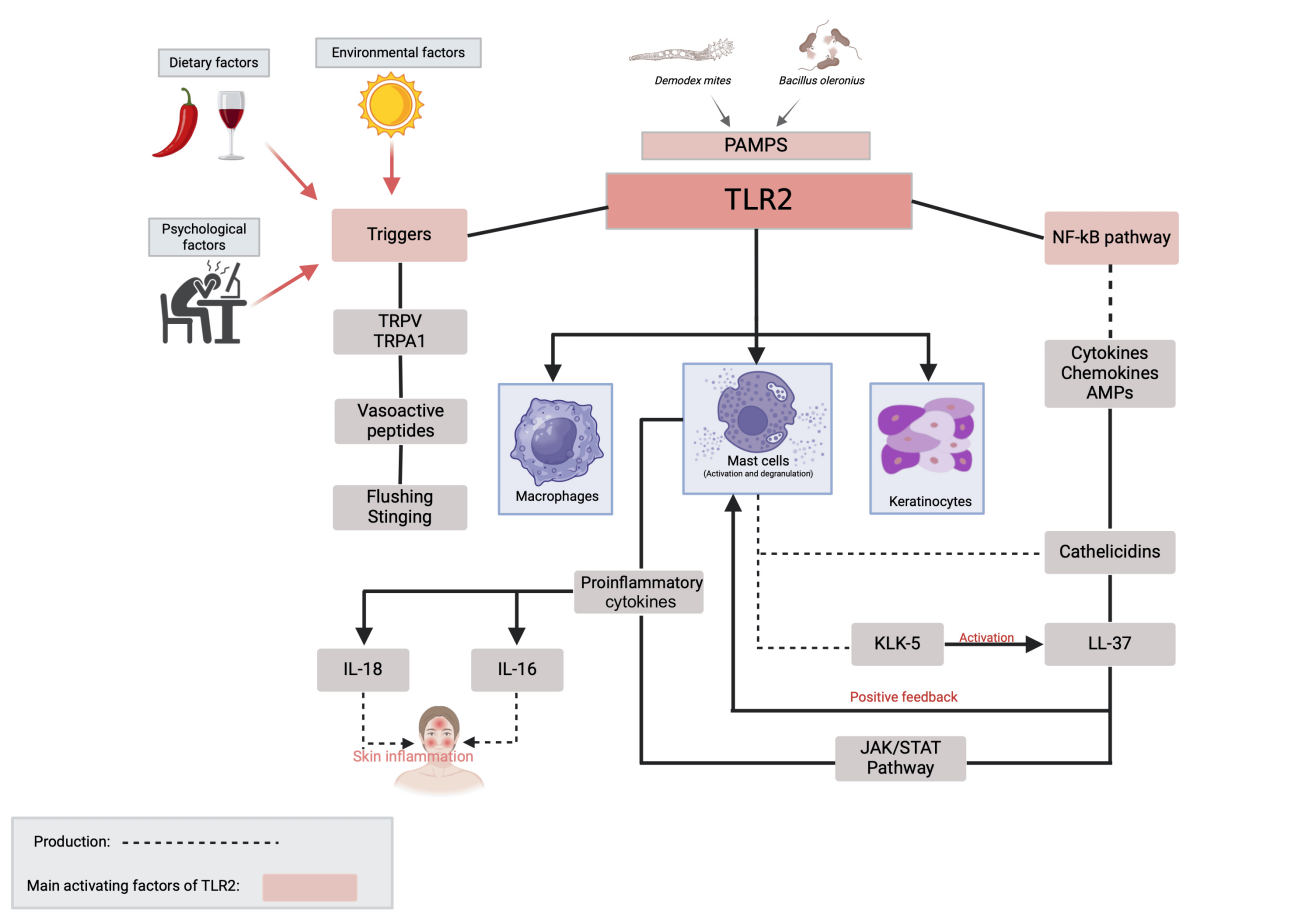
Pathophysiology of rosacea Schematic representation of the pathophysiology of rosacea The main triggers of rosacea include PAMPs, as well as environmental, dietary, and psychological factors. These stimuli activate TLR2, expressed on macrophages, mast cells, and keratinocytes (shown in blue). TLR2 activation initiates the NF-κB signaling pathway, resulting in the production of chemokines, cytokines, and AMPs. Among these, the cathelicidin LL-37 requires activation by the serine protease KLK-5, both of which are primarily derived from mast cells. LL-37 further stimulates mast cells, driving chemotaxis, degranulation, and pro-inflammatory cytokine release, thereby creating a positive feedback loop. Additionally, LL-37 activates the JAK/STAT pathway, amplifying cytokine release and sustaining cutaneous inflammation. Beyond immune activation, environmental, nutritional, and emotional factors also contribute to neurovascular hyperreactivity by stimulating TRPV1 and TRPA1 channels. This leads to the release of vasoactive peptides, which promote flushing and stinging sensations. PAMPS: pathogen-associated molecular patterns, TLR2: Toll-like receptor 2, NF-κB: nuclear factor-kappa B, AMPs: antimicrobial peptides, JAK/STAT: Janus kinase/signal transducers and activators of transcription, TRPV1: transient receptor potential vanilloid 1, TRPA1: transient receptor potential ankyrin 1, KLK-5: kallikrein 5 Created by the authors using BioRender.com

The pathophysiology of rosacea is multifactorial and not yet fully understood, involving a complex interplay of genetic, environmental, immune, and neurovascular factors (Figure 1).

**Immune dysregulation and innate immunity

Immune dysregulation (innate, adaptive, and inflammasome) and impaired neurocutaneous signaling contribute to chronic inflammation [7], leading to the production of excessive inflammatory cytokines and antimicrobial peptides, including cathelicidin, kallikrein 5 (KLK-5), and LL-37 [8]. Overgrowth of commensal skin microorganisms further worsens inflammation [9]. Elevated reactive oxygen species (ROS) also play a key role, as increased ROS levels are found in patients with rosacea [10].

Role of skin microbiota and *Demodex*

The skin microbiota plays a central role in the pathogenesis of rosacea. While the microbiota refers to microbial cells residing on the skin, the microbiome also includes their genetic material [11]. This ecosystem is highly dynamic and influenced by environmental, host, and lifestyle factors such as humidity, temperature, pH, diet, medications, and stress [12]. Under healthy conditions, a balanced skin microbiome supports barrier integrity, regulates immune responses, and suppresses pathogenic microorganisms. Disruption of this balance, through barrier dysfunction or dysbiosis, promotes cutaneous inflammation and contributes to rosacea severity [13].

Some authors have demonstrated that the skin microbiome of patients with rosacea differs significantly from that of healthy individuals. Changes in both alpha (diversity within an individual microbiome sample) and beta (assesses differences in microbial composition between samples or groups) diversity have been reported, along with increased relative abundances of genera such as *Staphylococcus*, *Corynebacterium*, *Cutibacterium*, and *Neisseria.* Beyond taxonomic differences, predicted metabolic pathways derived from the skin and gut microbiomes of patients with rosacea exhibit predominantly pro-inflammatory profiles, whereas those observed in rosacea-free individuals tend to exert anti-inflammatory effects, highlighting the functional relevance of microbial alterations [14].

Genetic and epidemiologic approaches further support a causal association between specific microorganisms and rosacea susceptibility. Mendelian randomization studies have linked certain bacterial taxa, including uncategorized *Staphylococcus *[15] and *Corynebacterium *species [16], to an increased risk of rosacea, while other taxa appear to confer a protective effect [17]. Although these studies provide limited mechanistic insight, they strengthen the evidence that microbial composition influences disease risk and expression.

At the mechanistic level, rosacea is closely associated with overgrowth or imbalance of commensal microorganisms such as *Demodex* *folliculorum*, *Bacillus* *oleronius*, *Staphylococcus* *epidermidis*, and *Cutibacterium* *acnes* [9,18]. Increased *Demodex* density is consistently observed across rosacea subtypes, and with significantly higher densities when compared with healthy controls [19-21]. *Staphylococcus* *epidermidis* has emerged as another relevant microbial contributor, as coagulase-negative staphylococci isolated from rosacea lesions secrete pro-inflammatory proteins [22] and display increased β-hemolytic activity at high temperature [23]. Together with *Bacillus* *oleronius*, these microorganisms act as microbial triggers that activate innate and adaptive immune responses [7], partly by enhancing interactions between microbial antigens and host immune cells [24-26]. Microbial pathogen-associated molecular patterns (PAMPs) activate Toll-like receptor 2 (TLR2) on keratinocytes, mast cells, and endothelial cells, triggering nuclear factor-kappa B (NF-κB)-mediated inflammatory cascades [3,26-28]. This leads to increased expression of KLK-5 and aberrant processing of cathelicidin into LL-37, which amplifies inflammation through mast cell degranulation, cytokine release, angiogenesis, and modulation of UV-induced inflammatory responses, further contributing to rosacea’s characteristic photosensitivity [29].

Neurovascular and neuroimmune mechanisms

Neurovascular dysregulation is a hallmark of rosacea and contributes to flushing, erythema, and sensory symptoms. Transient receptor potential channels (TRP), particularly TRPV1 (vanilloid) and TRPA1 (ankyrin), are overexpressed in rosacea and respond to thermal, chemical, and emotional triggers. Activation of these channels induces neurogenic inflammation through the release of vasoactive neuropeptides, linking environmental stimuli to immune and vascular responses [30,31].

Gut-skin axis and systemic inflammation

The skin is influenced not only by its resident microbiota but also by the gastrointestinal microbiome. Increasing evidence links gastrointestinal health to inflammatory skin conditions such as rosacea, in which gastrointestinal disorders are among the most frequently reported comorbidities [22]. Under physiological conditions, a diverse and stable gut microbiome functions as a barrier that limits the translocation of harmful microbial products into the systemic circulation. Disruption of gut microbial composition or intestinal barrier integrity may facilitate systemic inflammation, thereby affecting peripheral tissues, including the skin [27,32].

The concept of the gut-skin axis describes the bidirectional communication between the gastrointestinal tract and the skin through immunological, metabolic, and neuroendocrine pathways [33]. Maintenance of intestinal barrier integrity, supported by mucus, immune cells, IgA, and antimicrobial peptides, is essential for preserving skin homeostasis [34]. Large population-based studies strongly support this association; notably, a nationwide cohort study demonstrated significantly increased risks of celiac disease, inflammatory bowel disease, and irritable bowel syndrome among patients with rosacea [35]. These observations, together with accumulating evidence, have led to the proposal of an expanded gut-brain-skin axis, emphasizing shared inflammatory and neuronal pathways [35-38].

Mechanistically, gut dysbiosis may contribute to rosacea through immune activation and increased intestinal permeability, allowing bacterial components or DNA to enter the circulation and promote systemic inflammation [31,33,39,40]. Alterations in gut microbial composition have been associated with exaggerated immune responses [18], pro-inflammatory cytokine production, and activation of pathways such as the plasma kallikrein-kinin system, which may further drive neurogenic inflammation [41,42]. Although studies evaluating gut microbiome diversity in rosacea remain limited, consistent differences in microbial composition, particularly reduced abundance of short-chain fatty acid-producing bacteria, have been reported and may be relevant to both cutaneous inflammation and gastrointestinal symptoms [38,43-45].

The potential involvement of extracutaneous microorganisms such as *Helicobacter* *pylori* and small intestinal bacterial overgrowth (SIBO) has also been explored, although findings remain heterogeneous [46,47]. While evidence supporting a direct pathogenic role of *H. pylori* in rosacea is inconsistent [34,48-51], SIBO appears more prevalent in selected rosacea populations [47,52,53], and its eradication has been associated with clinical improvement in some patients [52].

Given the close interaction between the skin and gut microbiome and immune dysregulation in rosacea, therapeutic strategies targeting the skin and gut microbiome have gained increasing attention. In this context, microbiome-modulating interventions, collectively referred to as biotics, represent a promising and evolving area of rosacea management.

Methods

A narrative literature review was performed to evaluate the role of microbiota-modulating therapies in rosacea. A search of the PubMed/MEDLINE database was conducted to identify relevant articles published between January 1999 and July 2025. Complementary searches were performed in Scopus and Web of Science to ensure broader coverage of dermatology and microbiome-related literature.

The search strategy included combinations of the following keywords and MeSH terms: “rosacea,” “gut-skin axis,” “microbiota,” “microbiome,” “probiotics,” “prebiotics,” “postbiotics,” and “synbiotics.” Additional terms related to microbial dysbiosis associated with rosacea, including “*Demodex*,” “*Helicobacter* *pylori*,” and “small intestinal bacterial overgrowth,” were also considered.

Eligible publications included randomized clinical trials, observational studies, case reports, and relevant preclinical or mechanistic studies addressing microbiota modulation in rosacea. Reviews were also considered when they provided relevant background on the gut-skin axis or microbiome involvement in inflammatory skin diseases. Editorials, duplicate publications, and studies not directly related to rosacea were excluded. Additional relevant studies were identified through manual screening of reference lists of selected publications.

## Review

Biotics in rosacea

In this review, the broader term “biotics” is used as an umbrella concept encompassing probiotics, prebiotics, postbiotics, and synbiotics, all of which aim to modulate the gut-skin axis and its downstream immunological and inflammatory effects [8,12]. Increasing evidence suggests that patients with rosacea exhibit alterations in both skin and gut microbial composition, frequently characterized by reduced microbial diversity and functional imbalance [18,31,38,39,41,43,44]. These findings support the hypothesis that microbial dysbiosis may contribute to disease pathogenesis through activation of inflammatory pathways, disruption of epithelial barrier integrity, and dysregulation of innate immune responses. Consequently, microbiome-targeted interventions have emerged as potential adjunctive strategies for the management of rosacea and other inflammatory skin disorders [14,17]. However, most of the currently available clinical evidence in rosacea focuses on probiotic interventions, whereas data regarding prebiotics, postbiotics, and synbiotics remain comparatively limited.

Probiotics

Probiotics are live microorganisms that, when administered in adequate amounts, confer health benefits to the host, primarily by promoting a balanced gut microbial environment [54,55]. These microorganisms can be delivered through dietary supplements, pharmaceutical formulations, or functional foods [34]. In rosacea, probiotics have been investigated for their ability to modulate both gut and skin microbiota, enhance skin barrier function, and regulate inflammatory signaling pathways such as TLR2 and nuclear factor kappa B (NF-κB) [12,56]. Through modulation of the gut-skin axis, probiotics may influence systemic and cutaneous immune responses, reduce neurogenic inflammation, and improve epithelial barrier integrity, thereby contributing to decreased inflammation and improved skin homeostasis in rosacea. Both oral and topical probiotic formulations have been explored as adjunctive therapeutic approaches, with several studies reporting improvements in erythema, inflammatory lesions, and skin sensitivity, as well as improvements in patient quality of life [3,8,9,55,57].

Clinical studies evaluating probiotics in rosacea remain relatively limited and are often characterized by small sample sizes and heterogeneous methodologies. Nevertheless, several investigations have explored probiotic supplementation as an adjunctive therapy, including strains such as *Escherichia coli *Nissle and combinations of *Bifidobacterium *and *Lactobacillus *spp., which have been associated with improved clinical outcomes and higher remission rates when used alongside conventional treatments [55,58-61]. A summary of key probiotic studies in rosacea is provided in Table 1.

**Table 1:** Clinical and preclinical evidence on biotic interventions in rosacea *Standard of care included seven days of oral antibiotics, vitamins, antihistamines, and topical permethrin. **Daily dose of 2 g mixed probiotic containing *Lacticaseibacillus paracasei *Zhang, *Lactiplantibacillus plantarum *P-8, *Lacticaseibacillus rhamnosus *Probio-M9, *Bifidobacterium animalis *subsp. *lactis* V9, and Probio-M8. ***Strains selected based on comparative fecal microbiota analysis between healthy controls and patients with rosacea, demonstrating acid resistance, antioxidant capacity, antimicrobial tolerance, and inhibition of pathogenic bacterial growth. ****Formulation included Vichy volcanic mineralizing water, probiotic fractions of *Vitreoscilla filiformis*, hyaluronic acid, niacinamide, and tocopherol. IgA: immunoglobulin A, IL-8: interleukin-8, IFNα: interferon-alpha, KLK-5: kallikrein 5, TNF-α: tumor necrosis factor-alpha, NF-κB: nuclear factor-kappa B

Author, year	Study design	Population/model	Biotic intervention	Comparator	Main outcomes assessed	Key findings
Fortuna et al. (2016) [59]	Case report	Adult patient with papulopustular rosacea and ocular involvement	Oral probiotics (*Bifidobacterium breve* BR03, *Lactobacillus salivarius* LS01) plus doxycycline (40 mg/day) for 8 weeks, followed by probiotic monotherapy for 6 months	Not applicable	Clinical response and disease relapse	Marked clinical improvement with sustained remission after antibiotic withdrawal
Manzhalii et al. (2016) [60]	Randomized, controlled, non-blinded clinical trial	57 patients with facial inflammatory dermatoses, including papulopustular rosacea	Oral *Escherichia coli* Nissle 1917 plus standard topical therapy and vegetarian diet	Standard topical therapy and vegetarian diet	Clinical response, quality of life, immune markers (IgA, IL-8, and IFNα), stool characteristics, and gut microbiota composition	Higher remission rates, normalized IgA, reduced IL-8, favorable microbiota shifts, normalized stool consistency, improved quality of life; no adverse events
Buianova et al. (2018) [58]	Randomized clinical trial	60 adults with rosacea	*Bifidobacterium*-containing probiotic plus Polyoxidonium (thrice daily) for 21 days plus standard of care*	Standard of care*	Clinical remission and gut microbiota	Higher remission rates and restoration of *Lactobacillus* and *Bifidobacterium* populations
Yu et al. (2024) [55]	Randomized, double-blind, placebo-controlled trial	60 adults with rosacea	Multistrain oral probiotics for 3 months following a 2-week doxycycline (50 mg twice daily) course	Placebo or no further treatment	Physician global assessment score, skin parameters (hydration, sebum, pH), cytokines (IL-8, KLK-5, TNF-α), skin and gut microbiota, and antibiotic resistance genes	Improved severity, reduced inflammation, enhanced skin hydration, increased beneficial microbiota in the face and gut, and reduced antibiotic resistance genes
Qi et al. (2024) [61]	Preclinical translational study	LL-37-induced rosacea-like mouse model	*Lactobacillus salivarius* 23-006 and *Lactobacillus paracasei* 23-008 for 2 weeks	Untreated model	Inflammation, immune signaling, gut barrier integrity	Reduced erythema and inflammation, suppressed TLR2/NF-κB signaling, restored gut barrier
Berardesca et al. (2023) [62]	Randomized, split-face controlled clinical trial	20 adults with erythematous rosacea and sensitive skin wearing face masks	Topical *Vitreoscilla filiformis* probiotic fractions for 30 days	Contralateral untreated side	Erythema, transepidermal water loss, *Demodex* density, and tolerability	Reduced erythema and sensitivity, improved barrier function, high tolerability

Fortuna et al. reported a case of a 37-year-old patient with papulopustular rosacea involving the forehead and scalp and associated with ocular manifestations who was treated with low-dose doxycycline combined with oral probiotics (*Bifidobacterium breve *BR03 and *Lactobacillus salivarius* LS01). After eight weeks of treatment, marked improvement in both cutaneous and ocular symptoms was observed, and sustained remission was maintained for six months following antibiotic discontinuation and continued probiotic therapy [59].

Manzhalii et al. conducted a randomized, controlled but non-blinded clinical trial including 57 patients with inflammatory facial dermatoses characterized by erythema and papulopustular lesions, including rosacea, to evaluate oral supplementation with *Escherichia coli* Nissle 1917. Patients receiving probiotic therapy in addition to standard treatment demonstrated significantly higher rates of clinical improvement or complete remission compared with controls (89% versus 56%, P < 0.01), along with reductions in erythema and inflammatory lesions. Improvements were also observed in microbiological and immunological parameters, including normalization of serum IgA levels and decreased IL-8 concentrations [60].

Similarly, Buianova et al. conducted a randomized clinical trial involving 60 adult patients with rosacea comparing standard therapy alone with standard therapy plus a probiotic formulation containing *Bifidobacterium* species and the immunomodulator Polioxidonium. Patients receiving the probiotic-based regimen demonstrated higher rates of remission and clinical improvement. Notably, patients with rosacea initially exhibited reduced intestinal levels of *Lactobacillus* and *Bifidobacterium *compared with healthy individuals, which increased following probiotic supplementation, suggesting a potential link between microbiota modulation and clinical outcomes [58].

More recently, Yu et al. performed a randomized, double-blind, placebo-controlled study in 60 patients with rosacea to evaluate multistrain probiotic supplementation following a standardized course of doxycycline. Participants receiving probiotics showed greater clinical improvement, including reductions in inflammatory markers such as interleukin-8 (IL-8) and tumor necrosis factor-alpha (TNF-α), improved skin hydration, and favorable changes in both gut and facial microbiota composition. Additionally, probiotic supplementation was associated with a reduction in antibiotic resistance genes within the gut microbiome, suggesting a potential role in microbiome restoration after antibiotic therapy [55].

Experimental and translational research has further explored the mechanistic effects of probiotics in rosacea. Qi et al. investigated the therapeutic potential of* Lactobacillus salivarius *23-006 and* Lactobacillus paracasei *23-008 in a murine model of LL-37-induced rosacea-like inflammation. Oral administration of these strains significantly reduced erythema and inflammatory cytokine expression while suppressing the TLR2/MyD88/NF-κB signaling pathway. These effects were accompanied by restoration of gut microbiota balance and improved intestinal barrier integrity, supporting the concept that modulation of the gut-skin axis may influence rosacea pathophysiology [61].

Topical probiotics have also been investigated. Berardesca et al. conducted a randomized split-face clinical trial in 20 patients with erythematous rosacea using a dermocosmetic formulation containing probiotic fractions of *Vitreoscilla filiformis*. Treatment resulted in improvements in erythema, skin sensitivity, hydration, and transepidermal water loss, along with a reduction in *Demodex *density. The product was well tolerated and associated with high patient satisfaction [62].

Despite these promising findings, the available literature on probiotics in rosacea remains heterogeneous with respect to probiotic strains, dosing regimens, study design, outcome measures, and treatment duration. Consequently, direct comparison between studies is challenging, and the identification of optimal probiotic formulations for rosacea remains unresolved. In addition, some reports suggest that the beneficial effects of probiotic supplementation may diminish after treatment discontinuation, indicating that sustained or repeated administration may be required to maintain clinical benefits [62].

Prebiotics

Prebiotics are non-digestible substrates that selectively stimulate the growth and metabolic activity of beneficial microorganisms within the intestinal microbiota [8]. Through fermentation by gut bacteria, prebiotics promote the production of short-chain fatty acids and other metabolites that contribute to intestinal barrier integrity, immune regulation, and anti-inflammatory effects [63]. These metabolites, particularly short-chain fatty acids such as butyrate, have been shown to modulate immune responses, enhance epithelial barrier function, and influence systemic inflammatory pathways.

These mechanisms are relevant to the gut-skin axis and may theoretically influence inflammatory skin conditions such as rosacea. Alterations in gut microbial composition observed in patients with rosacea suggest that interventions capable of restoring microbial balance could potentially reduce systemic inflammation and improve skin homeostasis. By promoting the growth of beneficial bacterial taxa, prebiotics may indirectly modulate immune responses and contribute to the restoration of microbial equilibrium within the gut microbiome.

However, clinical studies specifically evaluating prebiotic supplementation in rosacea are currently lacking. Most available evidence derives from broader research on microbiota modulation or from studies conducted in other inflammatory dermatoses, including atopic dermatitis [34]. While these findings suggest that prebiotics may help regulate systemic immune responses and maintain epithelial barrier homeostasis, their direct role in rosacea remains largely theoretical. The absence of targeted clinical investigations highlights an important gap in current research and underscores the need for well-designed clinical trials to determine whether prebiotic interventions could provide therapeutic benefit in rosacea.

Postbiotics

Postbiotics are defined as preparations of inanimate microorganisms or their bioactive components that confer health benefits to the host [64]. These preparations may include microbial metabolites, cell wall components, enzymes, peptides, and other biologically active molecules produced during microbial fermentation. Unlike probiotics, postbiotics do not contain viable microorganisms, which may offer advantages in terms of safety, stability, and standardization of therapeutic formulations.

Recent research has highlighted the potential role of postbiotics in modulating inflammatory pathways associated with the gut-skin axis. Microbial metabolites and structural components derived from beneficial bacteria have been shown to influence host immune responses, regulate epithelial barrier integrity, and modulate signaling pathways involved in inflammatory skin diseases. These mechanisms may be particularly relevant in rosacea, where dysregulated innate immune responses and abnormal inflammatory signaling contribute to disease pathogenesis.

Emerging experimental evidence suggests that postbiotic compounds derived from *Lactobacillus *species may influence inflammatory pathways relevant to rosacea. For instance, postbiotics derived from *Lactobacillus salivarius* 23-006 and *Lactobacillus paracasei *23-008 have demonstrated inhibitory effects on LL-37-induced activation of the NF-κB pathway and downregulation of inflammatory mediators such as KLK-5 in experimental models [61]. These compounds have also shown bacteriostatic activity against potential pathogenic microorganisms, including *Staphylococcus aureus.*

Although these findings provide mechanistic insights into the potential anti-inflammatory effects of postbiotics, current evidence in rosacea remains limited to experimental and preclinical studies. To date, clinical trials evaluating postbiotic therapy in rosacea have not yet been conducted, and further research is required to determine whether these compounds could translate into effective therapeutic strategies.

Synbiotics

Synbiotics are formulations that combine probiotics and prebiotics in order to produce synergistic effects on the gut microbiota [11]. By simultaneously introducing beneficial microorganisms and substrates that support their growth, synbiotics may enhance microbial colonization, metabolic activity, and overall microbiome stability [63]. Through these combined effects, synbiotics may theoretically influence the gut-skin axis by promoting microbial balance, modulating immune responses, and improving epithelial barrier integrity.

Clinical studies evaluating synbiotics have demonstrated beneficial effects in several inflammatory and allergic skin diseases, particularly atopic dermatitis [65,66]. These findings suggest that combined microbiota-targeted strategies may help regulate systemic inflammation and improve skin barrier function. However, to date, no clinical trials have specifically investigated synbiotic therapy in rosacea. Consequently, the potential role of synbiotics in rosacea management remains uncertain and represents an important area for future research.

Taken together, microbiota-targeted therapies represent a promising area of investigation in rosacea. While probiotics have been evaluated in several small clinical and experimental studies, evidence supporting the use of prebiotics, postbiotics, and synbiotics remains limited. The heterogeneity of available studies, small sample sizes, and variability in treatment protocols underscore the need for larger, well-designed randomized controlled trials to clarify the therapeutic potential of microbiome-modulating strategies in rosacea.

Discussion

Rosacea is a chronic inflammatory skin disorder with a multifactorial pathogenesis involving immune dysregulation, neurovascular alterations, and microbial imbalance. The transition from a subtype-based classification to a phenotype-based model has improved the clinical characterization of rosacea and enabled more individualized treatment strategies [6]. Increasing evidence highlights the role of microbial dysbiosis in both the skin and gastrointestinal tract, supporting the concept of the gut-skin axis as an important contributor to disease pathogenesis [22,27].

Alterations in innate immune signaling, particularly exaggerated Toll-like receptor (TLR) activation and increased expression of antimicrobial peptides such as LL-37, contribute to the inflammatory cascade observed in rosacea [3,7]. In addition, increased *Demodex* *folliculorum* density and overgrowth of certain bacterial species have been associated with inflammatory lesions [4,27]. These findings provide a biological rationale for microbiome-targeted therapies, including probiotics, prebiotics, postbiotics, and synbiotics.

Among these strategies, probiotics have been the most extensively investigated. Several clinical and experimental studies suggest that probiotic supplementation may modulate gut and skin microbiota, reduce inflammatory mediators, and improve skin barrier function. In small clinical trials, oral probiotic administration has been associated with reductions in inflammatory lesions and improvements in quality of life when used as adjunctive therapy alongside conventional treatments such as doxycycline [55,58-62]. However, probiotics have rarely been evaluated as standalone therapies, and their effects are often assessed in combination with antibiotics, dietary interventions, or topical treatments.

Critical appraisal of current evidence

Despite these promising findings, the available evidence regarding biotic therapies in rosacea remains limited and heterogeneous. One major limitation is the substantial variability in study design across available trials. Differences in probiotic strains, dosing regimens, treatment duration, and outcome measures make direct comparison between studies difficult and limit the ability to establish standardized therapeutic recommendations.

Another important limitation is the predominance of small, short-term studies. Most clinical trials involve relatively small sample sizes and follow-up periods ranging from several weeks to a few months. Consequently, the long-term efficacy and safety of probiotic therapy in rosacea remain unclear, particularly with regard to relapse prevention after treatment discontinuation.

In addition, probiotic effects appear to be strain-specific, and the selection of probiotic strains in current studies is highly variable. This heterogeneity complicates the interpretation of the available evidence and highlights the need for more standardized approaches to probiotic selection and formulation. Furthermore, few studies include rigorous methodological features such as double-blinding, adequate placebo controls, or formal assessment of microbiome composition before and after treatment.

Evidence supporting other microbiota-targeted therapies remains even more limited. While prebiotics may theoretically influence the gut-skin axis by promoting beneficial microbial growth and increasing short-chain fatty acid production, clinical studies specifically evaluating prebiotic supplementation in rosacea are currently lacking. Similarly, postbiotics and synbiotics have demonstrated immunomodulatory effects in experimental models and other inflammatory skin diseases, but their role in rosacea has not yet been evaluated in controlled clinical trials.

Clinical implications and future directions

Given these limitations, microbiome-targeted therapies should currently be considered adjunctive rather than primary treatments for rosacea. While probiotics may offer potential benefits in selected patients, particularly when used alongside standard therapies, the available evidence remains insufficient to support routine clinical recommendations.

Future research should focus on well-designed randomized controlled trials with larger patient populations, longer follow-up periods, and standardized outcome measures. In addition, advances in microbiome sequencing technologies may allow for more precise characterization of microbial dysbiosis in rosacea and facilitate the development of personalized microbiota-targeted therapies.

## Conclusions

In conclusion, microbiota-targeted therapies represent an emerging area of interest in rosacea research. Current evidence suggests that probiotics may provide potential benefits as adjunctive treatments through modulation of the gut-skin axis and inflammatory pathways. However, available studies remain limited and heterogeneous, and evidence supporting the role of prebiotics, postbiotics, and synbiotics in rosacea is still scarce. Consequently, well-designed randomized controlled trials with standardized methodologies and longer follow-up periods are required before microbiome-modulating strategies can be incorporated into evidence-based clinical practice.
